# Different types of FC **γ** -receptors are involved in anti-Lewis Y antibody induced effector functions in vitro

**DOI:** 10.1054/bjoc.1999.0940

**Published:** 2000-01-18

**Authors:** M Dettke, H Loibner

**Affiliations:** Clinic for Blood Group Serology and Transfusion Medicine, University Hospital of Vienna, Austria; NOVARTIS Forschungsinstitut, Vienna, Austria

**Keywords:** monocytes, anti-LeY antibody, FcγR, ADCC, TNF-α

## Abstract

Stimulation of monocytes by interaction of monoclonal antibodies (mAbs) with Fc gamma receptors (FcγRs) results in the activation of various monocyte effector functions. In the present investigation we show that the anti-Lewis Y (LeY) anti-tumour mAb ABL 364 and its mouse/human IgG1 chimaera induce both antibody-dependent cellular cytotoxicity (ADCC) and the release of tumour necrosis factor α (TNF-α) during mixed culture of monocytes with LeY^+^SKBR5 breast cancer cells in vitro. Although anti-LeY mAb-mediated TNF-α release paralleled ADCC activity, cytokine release required a higher concentration of sensitizing mAb than the induction of cytolysis. The determination of the FcγR classes involved in the induction of the distinct effector functions showed that anti-LeY mAb-induced cytolysis was triggered by interaction between anti-LeY mAbs and FcγRI. In contrast, mAb-induced TNF-α release mainly depended on the activation of monocyte FcγRII. Neutralization of TNF-α showed no influence on monocyte ADCC activity towards SKBR5 target cells. Our data indicate an independent regulation of anti-LeY mAb induced effector functions of ADCC and TNF-α release which seemed to be triggered by activation of different types of FcγR. © 2000 Cancer Research Campaign


*British Journal of Cancer* (2000) **82**(2), 441–445
© 2000 Cancer Research Campaign
Article no. bjoc.1999.0940

### Different types of FCγ-receptors are involved in anti-Lewis Y antibody induced effector functions in vitro

**M Dettke[1,2] and H Loibner[2]**

[1]Clinic for Blood Group Serology and Transfusion Medicine, University Hospital of Vienna, Austria; [2]NOVARTIS Forschungsinstitut, Vienna, Austria

**Summary** Stimulation of monocytes by interaction of monoclonal antibodies (mAbs) with Fc gamma receptors (FcγRs) results in the activation of various monocyte effector functions. In the present investigation we show that the anti-Lewis Y (LeY) anti-tumour mAb ABL 364 and its mouse/human IgG1 chimaera induce both antibody-dependent cellular cytotoxicity (ADCC) and the release of tumour necrosis factor α (TNF-α) during mixed culture of monocytes with LeY$^+$ SKBR5 breast cancer cells in vitro. Although anti-LeY mAb-mediated TNF-α release paralleled ADCC activity, cytokine release required a higher concentration of sensitizing mAb than the induction of cytolysis. The determination of the FcγR classes involved in the induction of the distinct effector functions showed that anti-LeY mAb-induced cytolysis was triggered by interaction between anti-LeY mAbs and FcγRI. In contrast, mAb-induced TNF-α release mainly depended on the activation of monocyte FcγRII. Neutralization of TNF-α showed no influence on monocyte ADCC activity towards SKBR5 target cells. Our data indicate an independent regulation of anti-LeY mAb induced effector functions of ADCC and TNF-α release which seemed to be triggered by activation of different types of FcγR. © 2000 Cancer Research Campaign

**Keywords:** monocytes; anti-LeY antibody; FcγR; ADCC; TNF-α

The monoclonal antibody (mAb) ABL 364 is a murine mAb of the IgG3 subclass specific for the Lewis Y (LeY) carbohydrate antigen, an oncofetal oligosaccharide frequently expressed on epithelial tumour cells (Blaszczyk-Thurin et al, 1987; Steplewski et al, 1990). ABL 364 induces a profound anti-tumour response by activation of both, complement-dependent tumour cell cytotoxicity (CDC) and human effector cells for antibody-dependent cellular tumour cell destruction (ADCC) (Scholz et al, 1991). Preliminary data of current clinical trials (phase I/II) indicate that mAb ABL 364 exerts a clinical benefit in vivo, especially in patients with minimal residual cancer disease (Schlimok et al, 1995). One drawback in the use of mAb ABL 364 for therapeutic purposes is the development of a human anti-mouse response which can render them ineffective for repeat therapy. To avoid this disadvantage, parietal and fully humanized isotypes from the murine ABL 364 have been constructed (Co et al, 1996).

In ADCC, a common characteristic of effector cells is the expression of receptors for the Fc portion of IgG (FcγR). Freshly isolated human monocytes constitutively express two different classes of human FcγRs: the high affinity receptor FcγRI (CD64) and the low affinity receptor FcγRII (CD32) (Deo et al, 1997). Apart from their function as trigger molecules in antibody-mediated cellular cytolysis (Van de Winkel et al, 1989), both classes of FcγR have been implicated in Fc-dependent stimulation of cytokine release (Debets et al, 1990). Signalling through FcγRs activates human leucocytes to secret various pro-inflammatory cytokines like GM-CSF (Herrmann et al, 1992), interleukin (IL)-6 (Ling et al, 1990) or IL-8 (Marsh et al, 1995).

*Received 4 January 1999*
*Revised 26 July 1999*
*Accepted 2 August 1999*

*Correspondence to:* M Dettke, Clinic for Blood Group Serology and Transfusion Medicine, AKH Wien, Währinger Gürtel 18–20, A-1090 Vienna, Austria

While in most of the previous reports FcγR-triggered cytokine release was induced either by solid-phase bound IgG (Herrmann et al, 1992), complexed IgG (Ling et al, 1990) or IgG-sensitized erythrocytes (Davenport et al, 1994), there is little known about the FcγR-mediated cytokine release induced by anti-tumour antibodies bound to their tumour cell-associated antigens (Wing et al, 1996). This may be of importance in the preclinical evaluation of anti-tumour mAbs. The specific release of cytokines at the site of the tumour may enhance the local inflammatory response and thus may interfere with the cytotoxic properties of attached immunocytes (Bonta and Ben Efraim, 1993).

In view of the clinical use of mAb ABL 364 and related anti-LeY mAbs in the treatment of cancer, we investigated the release of TNF-α in anti-LeY mAb-mediated ADCC in vitro. Additionally we were interested in the subclasses of FcγR involved in either mAb-stimulated monokine release or anti-LeY mAb-triggered cytotoxic response.

### MATERIALS AND METHODS

### Anti-LeY antibodies

The characterization and purification of anti-LeY mAb ABL 364 and its mouse/human IgG1 chimaera has been described (Blaszczyk-Thurin et al, 1987; Co et al, 1996).

### Isolation and culture of human effector cells

Enriched peripheral blood monocytes were isolated from the peripheral blood of healthy donors by centrifugal elutriation. Purity was > 90% as assessed by FACS analysis using a monoclonal antibody against CD14 (Becton Dickinson, USA). Human blood cells were cultured in RPMI-1640 supplemented with 10% fetal calf serum (FCS; Sigma, USA). To inhibit any effect of residual lipopolysaccharide (LPS), 10 μg $ml^{-1}$ polymyxin B was added to the culture medium (Sigma, USA).

### Tumour cell line

The human breast cancer cell line SKBR5 was used as $LeY^+$ target cells. The cell line was obtained from the Wistar Institute (Philadelphia, PA, USA).

### Preparation of SKBR5 membrane vesicles

Membrane vesicles of SKBR5 tumour cells were prepared according to the method described (Thom et al, 1977). For removal of membrane-bound glycodeterminants, the membrane preparation was sequentially digested with *N*-specific glycosidase (*N*-glycosidase F), neuraminidase (acylneuraminyl hydrolase) followed by endo-α-*N*-acetylgalactosaminidase to hydrolyse *O*-linked oligosaccharides (all enzymes from Genzyme, USA). For control, a LeY-bearing neoglycoprotein (LeY-BSA conjugate) was used (Chembiomed, Canada). Enzyme-induced hydrolysis of ABL 364-reactive LeY epitopes was monitored by enzyme-linked immunosorbent assay (ELISA). Unmodified membrane vesicles, enzyme-treated vesicles and controls (LeY-BSA, enzyme-treated LeY-BSA) were coated on microtitre plates. Following incubation with mAb ABL 364, bound ABL 364 was detected by horse-radish peroxidase-conjugated goat anti-mouse IgG3 (Southern Biotechnology, UK). Compared to unmodified membrane vesicles enzyme treatment reduced the presence of ABL364-reactive LeY epitopes on the membrane preparation by 80% (data not shown).

### In vitro ADCC assay

Anti-LeY mAb-induced ADCC was determined in a $^{51}$Cr-release assay as described previously (Co et al, 1996). SKBR5 tumour target cells were labelled with 100 μCi sodium ($^{51}$Cr) chromate (Amersham, UK) and incubated with monocytes at an effector:target (E/T) ratio of 15:1 in presence of anti-LeY mAbs or irrelevant isotype controls (mIgG3 or huIgG1; Sigma, USA). In selective experiments a polyclonal rabbit anti-human TNF-α preparation was added (Endogen, USA). After an overnight incubation, the radioactive supernatants were harvested and counted. The percentage of mAb-induced cytotoxicity was calculated according the formula:

$$\% \text{ lysis} = (a-b)/(c-b) \times 100$$

where a = radioactivity in supernatants from tested samples (cpm), b = spontaneous $^{51}$Cr release (cpm) in absence of effector cells and c = maximal release (cpm) after lysis of target cells with 2% sodium dodecyl sulphate (SDS). The mean percentage of mAb-induced specific lysis was determined from triple cultures. For generation of TNF-α-conditioned medium, monocytes were incubated at a E/T ratio of 15:1 with non-radiolabelled tumour cells. Overnight supernatants were collected, filtered through a 0.22 μm filter to remove any cell debris, and kept at –70°C.

For FcγR inhibition, anti-FcγRI mAb 197 and the F(ab)′2 fragment of anti-FcγRII mAb IV.3 were used (both Medarex, USA). The specificity of both anti-FcγR mAbs to their target structures has been described (Petroni et al, 1988; Guyre et al, 1989). Monocytes were preincubated for 30 min (4°C) with saturating concentrations (20 μg $ml^{-1}$) of the respective anti FcγR mAbs. After washing to remove unbound anti-FcγR mAbs, ADCC was performed as described.

### Determination of TNF-α

Immunoreactive TNF-α in the supernatants of ADCC cultures was determined by ELISA according to the manufacturer's protocol (Genzyme, USA). Each sample was tested in duplicate and mean values were calculated.

### Statistical analysis

Data were analysed for significance at $P < 0.05$ using the Student's *t*-test.

### RESULTS

Anti-LeY mAb-induced monocyte ADCC and TNF-α release in response to $LeY^+$ SKBR5 tumour target cells are shown in Figure 1. The addition of both mAbs to mixed effector/target cell cultures dose-dependently induced ADCC which was accompanied by a dose-dependent release of TNF-α. For both effector functions the chimaeric mAb was more potent than the murine IgG3 isotype. At 10 μg $ml^{-1}$ the IgG1 chimaera induced a cytotoxicity of 71 ± 14% compared to 48 ± 16% induced by mAb ABL 364 (Figure 1 A). A similar pronounced activity of the chimaera was also observed for TNF-α release (Figure 1 B). The F(ab)′2 fragment common for both mAbs failed to induce any ADCC or TNF-α release, demonstrating the Fc dependency of the observed effector functions.

When comparing the dose–response kinetics for both effector functions, antibody-induced TNF-α release required an approximately twofold higher concentration of sensitizing mAb than the induction of ADCC (Figure 2). While half maximal stimulation of cytotoxicity required the addition of approximately 4 μg $ml^{-1}$ of

**Table 1** Anti-LeY induced TNF-α release in response to SKBR5 membrane vesicles

| | mAb ABL 364 (μg $ml^{-1}$) | | | | IgG1 chimaeric mAb (μg $ml^{-1}$) | | | |
|---|---|---|---|---|---|---|---|---|
| | 0 | 5 | 10 | 20 | 0 | 5 | 10 | 20 |
| SKBR5 membrane vesicles | 45 ± 2 | 145 ± 17 | 230 ± 28 | 271 ± 57 | 61 ± 4 | 263 ± 28 | 342 ± 74 | 385 ± 92 |
| SKBR5 membrane vesicles after deglycosylation | 30 ± 6 | 59 ± 31 | 74 ± 17 | 63 ± 18 | 39 ± 13 | 72 ± 26 | 65 ± 19 | 56 ± 14 |

SKBR5 membrane vesicles or deglycosylated membrane vesicles (after sequential deglycosylsation with *N*-specific glycosidase, neuraminidase followed by *O*-specific glycosidase; both membrane vesicles at 6 μg $ml^{-1}$) were incubated with monocytes in the presence of increasing concentration of test antibodies. After an overnight incubation monocyte TNF-α release was determined. Data are expressed as mean ± s.e.m. ($n = 3$).

*© 2000 Cancer Research Campaign*

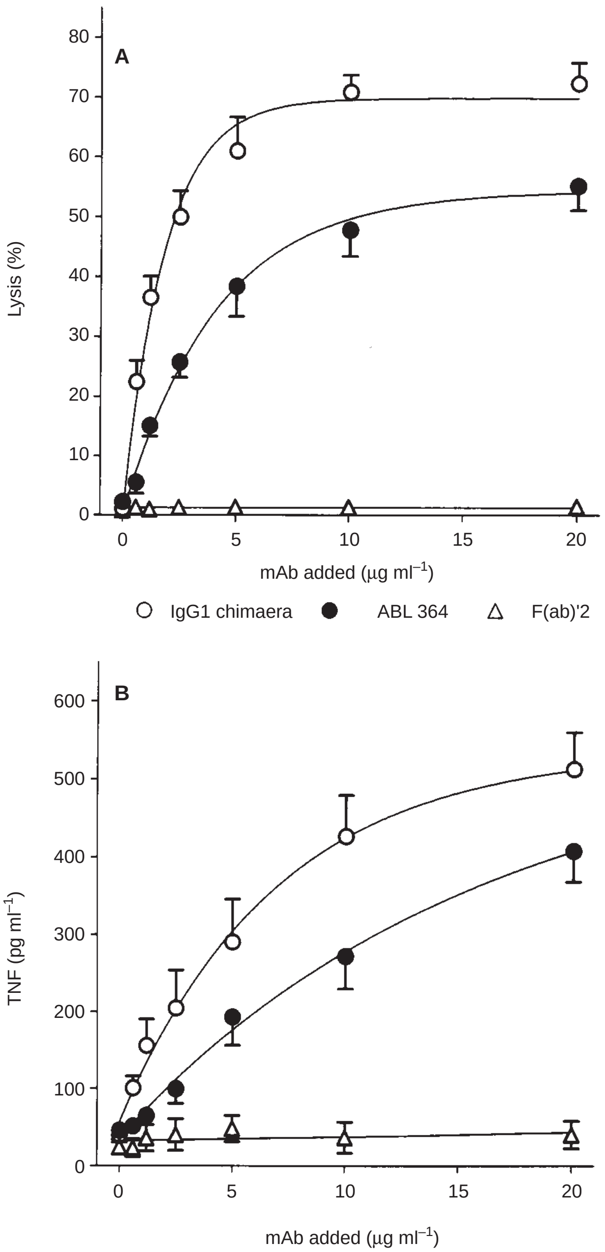

**Figure 1** Anti-LeY mAb-induced ADCC and TNF-α release in vitro. Monocytes were incubated at a E/T ratio of 15:1 with SKBR5 target cells in the presence of increasing concentration of test antibodies or their common F(ab)′2 fragment. (**A**) ADCC activity, (**B**) TNF-α release. Data are expressed as mean ± s.e.m. and represent the results of five experiments

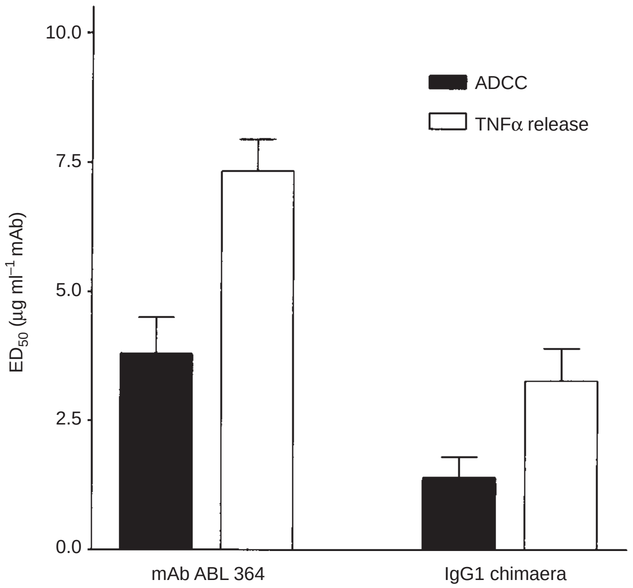

**Figure 2** Antibody concentration required for half-maximal ADCC and TNF-α release. [51]Cr labelled SKBR5 target cells were mixed with serial dilutions of test antibodies and monocytes at a E/T ratio of 15:1. The antibody concentration (mean ± s.e.m.) required for the induction of 50% of the maximal effector response ($ED_{50}$) are shown on the ordinate (*n* = 7 independent experiments)

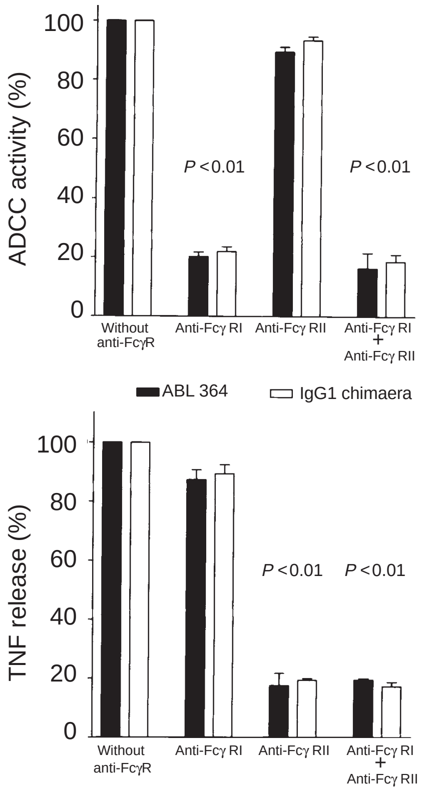

**Figure 3** Inhibition of anti-LeY mAb-induced effector functions after blocking of monocyte FcγRs. Prior to the ADCC assay monocytes were incubated with anti-FcγRI mAb 197 or F(ab)′2 anti FcγRII mAb IV.3 respectively. For induction of ADCC activity and TNF-α release test antibodies were used at a final concentration of 20 µg ml−1. Data (mean ± s.e.m.) are expressed as relative effector functions compared to the anti-LeY induced effector response without FcγR-blocking Abs (=100%)

mAb ABL 364 and 1.5 µg ml−1 of the IgG1 chimaera, half maximal release of TNF-α was achieved after addition of 7 µg ml−1 for mAb ABL 364 and 3 µg ml−1 for the chimaeric mAb.

To test whether the observed TNF-α release may be induced due to the release of cellular compounds of lytic tumour cells, we determined mAb-induced TNF-α release in the presence of LeY+ SKBR5 membrane vesicles instead of living target cells. In the presence of SKBR5 membrane vesicles both anti-LeY mAbs induced monocyte TNF-α release (Table 1). Removal of the LeY oligosaccharide from the membrane vesicles by sequential

© 2000 Cancer Research Campaign

**Table 2** Influence of anti-TNF-α Ab on anti-LeY mAb-induced monocyte ADCC

| | mAb ABL 364 (20 μg ml$^{-1}$) | IgG1 chimaeric mAb (20 μg ml$^{-1}$) |
|---|---|---|
| Without anti-TNF-α Ab | 54 ± 8% | 71 ± 6% |
| With anti-TNF-α Ab | 51 ± 4% | 65 ± 9% |

Anti-LeY mAb-induced monocyte ADCC against SKBR5 cells was performed in the presence of polyclonal anti TNF-α Ab. Data are expressed in % lysis (mean ± s.e.m.) and represent the results of three experiments.

deglycosylation with *N*-specific glycosidase, neuraminidase and *O*-specific glycosidase diminished mAb-induced TNF-α release (Table 1), therefore excluding the previously described stimulatory effect of tumour cell compounds on the TNF-α production as inducer of the observed cytokine release (Jänicke and Männel, 1990; DeMarco et al, 1992).

To characterize the type of monocyte FcγR involved in triggering anti-LeY mAb-induced effector functions, FcγR were sequentially blocked by mAbs directed against FcγRI or FcγRII. The preincubation of monocytes with anti-FcγRI mAb significantly reduced anti-LeY mAb-induced ADCC (Figure 3, upper panel). However, the blockade of FcγRI showed only a weak effect on the cytokine secretion, ranging from no inhibition to partial inhibition of TNF-α release (Figure 3, lower panel). In contrast, activation of FcγRII was not involved in anti-LeY-induced ADCC. While tumour cell destruction was only slightly diminished (<10%) after blocking of FcγRII, preincubation of monocytes with anti-FcγRII mAb reduced TNF-α release by 80% (Figure 3, lower panel). The combination of both anti-FcγR mAbs showed no additive effect in the inhibition of either anti-LeY mAb-induced ADCC or mAb-induced TNF-α release.

The lytic capacities of monocytes remained unchanged in the presence of anti-TNF-α antibodies. As shown in Table 2, neutralization of TNF-α showed no inhibitory effect on the anti-LeY mAb-induced cytolytic activity towards SKBR5 tumour cells.

### DISCUSSION

In the present study we showed that anti-LeY mAb ABL 364 and its mouse/human IgG1 chimaera are both capable of stimulating TNF-α release by human blood monocytes during condition of ADCC in vitro. Our data support and expand previous findings of an enhanced effector activity of the partial humanized mAb compared to the murine mAb (Co et al, 1996). Since the potential of an antibody to interact with FcR is determined in major part by the class of the constant region, this pronounced activity may be related to better interactions of monocytes with the chimaera bearing the human Fc compared to the murine IgG3 isotype (Shaw et al, 1988). However, irrespective of these quantitative differences in the effector response, both mAbs induced the distinct effector functions of ADCC and cytokine release through engagement of different subclasses of FcγRs. While anti-LeY mAb-induced ADCC activity depended on activation of FcγRI, stimulation of TNF-α release predominately depended on the engagement of FcγRII.

The FcγRII triggered TNF-α release required an approximately twofold higher concentration of anti-LeY mAb compared to the FcγRI-induced ADCC activity. This variation may be related to differences in the binding affinity of both mAbs to either FcγRI or FcγRII, i.e. the higher affinity of huIgG1 and mIgG3 to FcγRI compared to FcγRII (Lubeck et al, 1985). However, the specificity of distinct FcR subclasses for the various IgG isotypes is rather relative than absolute and seemed to be influenced by the density of bound antibodies on the target cell surface (Boot et al, 1989). At lower target cell sensitization anti-LeY mAb may therefore preferentially interact with FcγRI, and may lead to the observed cytotoxic response. This assumption is supported by previous data demonstrating that anti-LeY mAb-induced ADCC is diminished in the presence of human IgG (Scholz et al, 1991), presumably indicating a competition of monomeric IgG and anti-LeY mAbs for binding to FcγRI. However, with increased concentration of sensitizing mAb and subsequently, with an increased formation of a multivalent array of aggregated mAbs on the target cell surface, anti-LeY mAbs may additionally interact with FcγRII. It remains to be shown whether these effects might be related to a heterotypic cluster formation of both FcγR subtypes (Vossebeld et al, 1995), or due to the release of endogenous factors which have been shown to contribute to an up-regulation of FcγRII activity (Trevani et al, 1994; Salmon et al, 1995).

In the present study we did not test the influence of an FcγRIII blockade on anti-LeY mAb-mediated monocyte effector functions. On peripheral blood monocytes the expression of FcγRIII is restricted to a small subpopulation of monocytes (Deo et al, 1997). Although the expression of FcγRIII is up-regulated by various cytokines in vitro (Munn et al, 1991; Calzada-Wack et al, 1996), in our experiments at least 80% of the anti-LeY mAb-induced effector response was blocked by either anti-FcγRI or anti-FcγRII mAbs. Thus, a significant contribution of monocyte FcγRIII in activation of anti-LeY mAb-induced effector functions seems less likely.

Given that mAb ABL 364 and related anti-LeY mAbs induce a similar effector response in vivo, our finding that anti-LeY mAb-induced TNF-α release require a higher concentration of sensitizing mAb then the induction of cytotoxicity may have implications for the therapeutic application of both mAbs in vivo. Since TNF-α was not involved in mAb-induced cytotoxicity therefore confirming previous findings (Bungard et al, 1998), administration of low doses of anti-LeY mAb may lead to an effective anti-tumour response, even without induction of TNF-α release. This may minimize the risk of adverse reactions associated with a systemic anti-tumour antibody therapy, like the FcγR-triggered cytokine release syndrome (Wing et al, 1996; Tax et al, 1997). However, further clinical trials, particularly with the partial or fully humanized variant of mAb ABL 364, are warranted to evaluate the actual efficacy of an anti-LeY mAb-based immunotherapy on the overall survival time in cancer patients.

### ACKNOWLEDGEMENT

This work was supported by grant L 0033/ MED (M Dettke) from the ‘Österreichischen Fond zur Förderung der wissenschaftlichen Forschung’, Vienna, Austria.

© 2000 Cancer Research Campaign

### REFERENCES

Blaszczyk-Thurin M, Thurin J, Hindsgaul O, Karlsson KA, Steplewski Z and Koprowski H (1987) Y and blood group B type 2 glycolipid antigens accumulate in a human gastric carcinoma cell line as detected by monoclonal antibody. *J Biol Chem* **262**: 372–379

Bonta IL and Ben Efraim S (1993) Involvement of inflammatory mediators in macrophage antitumor activity. *J Leukoc Biol* **54**: 613–626

Boot JHA, Geerts MEJ and Aarden LA (1989) Functional polymorphisms of Fc receptors in human monocyte-mediated cytotoxicity towards erythrocytes induced by murine isotype switch variants. *J Immunol* **142**: 1217–1223

Bungard S, Flieger D, Schweitzer S, Sauerbruch T and Spengler U (1998) The combination of interleukin-2 and interferon alpha effectively augments the antibody-dependent cellular cytotoxicity of monoclonal antibodies 17-1A and BR55-2 against the colorectal carcinoma cell line HT29. *Cancer Immunol Immunother* **46**: 213–220

Calzada-Wack JC, Frankenberger M and Ziegler Heitbrock HWL (1996) Interleukin-10 drives human monocytes to CD16 positive macrophages. *J Inflamm* **46**: 78–85

Cassel DL, Keller MA, Surrey S, Schwartz E, Schreiber AD, Rappaport EF and McKenzie SE (1993) Differential expression of FcγRIIA, FcγRIIB and FcγRIIC in hematopoietic cells: analysis of transcripts. *Mol Immunol* **30**: 451–460

Co MS, Baker J, Bednarik K, Janzek E, Neruda W, Mayer P, Plot R, Stumper B, Vasquez M, Queen C and Loibner H (1996) Humanized anti-Lewis Y antibodies: in vitro properties and pharmacokinetics in rhesus monkeys. *Cancer Res* **56**: 1118–1125

Davenport RD, Burdick M, Strieter RM and Kunkel SL (1994) Monocyte chemoattractant protein production in red cell incompatibility. *Transfusion* **34**: 16–19

Debets JMH, Winkel van de JGJ, Ceuppens JL, Dieteren IEM and Buurman WA (1990) Cross-linking of both FcγRI and FcγRII induces secretion of tumor necrosis factor by human monocytes, requiring high affinity Fc-FcγR interactions. *J Immunol* **144**: 1304–1310

DeMarco R, Ensor JE and Hasday JD (1992) Tumor-stimulated release of TNF-α by human monocyte-derived macrophages. *Cell Immunol* **140**: 304–318

Deo YM, Graziano RF, Repp R and Winkel van de JGJ (1997) Clinical significance of IgG Fc receptor and FcγR-directed immunotherapies. *Immunol Today* **18**: 127–135

Guyre PM, Graziano RF, Vance BA, Morganelli PM and Fanger MW (1989) Monoclonal antibodies that bind to distinct epitopes on FcγRI are able to trigger receptor function. *J Immunol* **143**: 1650–1655

Herrmann F, Vos de S, Brach M, Riedel D, Lindemann A and Mertelsmann R (1992) Secretion of granulocyte-macrophage colony-stimulating factor by human blood monocytes is stimulated by engagement of Fcγ receptors type I by solid-phase immunoglobulins requiring high-affinity Fc-Fcγ receptor type I interactions. *Eur J Immunol* **22**: 1681–1685

Jänicke R and Männel DN (1990) Distinct tumor cell membrane constituents activate human monocytes for tumor necrosis factor synthesis. *J Immunol* **144**: 1144–1150

Ling ZD, Ziltener HJ, Webb BT and Matheson DS (1990) Aggregated immunoglobulin and Fc fragment of IgG induce IL6-release from human monocytes. *Cell Immunol* **129**: 95–103

Lubeck MD, Steplewski Z, Baglia F, Klein MH, Dorrington KJ and Koprowski H (1985) The interaction of murine IgG subclass proteins with human monocyte Fc receptors. *J Immunol* **135**: 1299–1304

Marsh CB, Gadek JE, Kindt GC, Moore SA and Wewers MD (1995) Monocyte Fcγ receptor cross-linking induces IL-8 production. *J Immunol* **155**: 3161–3167

Munn DH, McBride M and Cheung NKV (1991) Role of low affinity Fc receptors in antibody-dependent tumor cell phagocytosis by human monocyte-derived macrophages. *Cancer Res* **51**: 1117–1123

Petroni KC, Shen L and Guyre PM (1988) Modulation of human polymorphonuclear leukocyte IgG Fc receptors and Fc receptor-mediated functions by IFN-γ and glucocorticoids. *J Immunol* **140**: 3467–3472

Salmon JE, Millard SS, Brogle NL and Kimberly RP (1995) Fc gamma receptor IIIb enhances Fc gamma receptor IIa function in an oxidant-dependent and allele-sensitive manner. *J Clin Invest* **95**: 2877–2885

Schlimok G, Pantel K, Loibner H, Fackler-Schwalbe I and Rietmüller G (1995) Reduction of metastatic carcinoma cells in bone marrow by intravenously administered monoclonal antibody: towards a noval surrogate test to monitor adjuvant therapies of solid tumours. *Eur J Cancer* **31A**: 1799–1803

Scholz D, Lubeck M, Loibner H, McDonald-Smith J, Kimoto Y, Koprowski H and Steplewski Z (1991) Biological activity in the human system of isotype variants of oligosaccharide-Y-specific murine monoclonal antibodies. *Cancer Immunol Immunother* **33**: 153–157

Shaw DR, Kahazaeli MB and LoBuglio AF (1988) Mouse/human chimeric antibodies to a tumor-associated antigen: biologic activity of the four human IgG subclasses. *J Natl Cancer Inst* **80**: 1553–1559

Steplewski Z, Blaszczyk-Thurin M, Lubeck M, Loibner H, Scholz D and Koprowski H (1990) Oligosaccharide Y specific monoclonal antibody and its isotype switch variants. *Hybridoma* **9**: 201–210

Tax WJM, Frenken LA, Tamboer WPM, Jacobs CWM, Frenken LAM and Koene RAP (1997) Role of polymorphic Fc receptor FcγRIIa in cytokine release and adverse effects of murine IgG1 anti-CD3/T cell receptor antibody (WT31). *Transplantation* **63**: 106–112

Thom D, Powell AJ, Lloyd CW and Rees DA (1977) Rapid isolation of plasma membranes in high yield from cultured fibroblasts. *Biochem J* **168**: 187–194

Trevani AS, Andonegui GA, Isturiz MA, Schatner M, Serebrinsky G and Geffner JR (1994) Effect of proteolytic enzymes on neutrophil Fc gamma RII activity. *Immunology* **82**: 632–637

Vossebeld PJ, Kessler J, Borne von dem AE, Roos D and Verhoeven AJ (1995) Heterotypic Fc gamma R clusters evoke a synergistic $Ca^{2+}$ response in human neutrophils. *J Biol Chem* **270**: 10671–10679

Wing MG, Moreau T, Greenwood J, Smith RM, Hale G, Isaacs J, Waldmann H, Lachmann PJ and Compston A (1996) Mechanism of first-dose cytokine-release syndrome by CAMPATH 1-H: involvement of CD16 (FcγRIII) and CD11a/CD18 (LFA-1) on NK cells. *J Clin Invest* **98**: 2819–2826

Winkel van de JGJ, Boonen GJJC, Janssen LW, Vlug A, Hogg N and Tax WJM (1989) Activation of two types of Fc receptors, FcγI-R and FcγII-R, in human monocyte cytotoxicity to sensitized erythrocytes. *Scand J Immunol* **29**: 23–31

*© 2000 Cancer Research Campaign*

 *© 2000 Cancer Research Campaign*
